# Broadening Horizons: Exploring the Cathepsin Family as Therapeutic Targets for Alzheimer's Disease

**DOI:** 10.14336/AD.2024.0456

**Published:** 2025-06-02

**Authors:** Xiao-Hui Liu, Xiao-Tong Liu, Yue Wu, Shu-Ang Li, Kai-Di Ren, Meng Cheng, Bing Huang, Yang Yang, Pei-Pei Liu

**Affiliations:** ^1^Clinical Systems Biology Laboratories, the First Affiliated Hospital of Zhengzhou University, Zhengzhou, Henan, China.; ^2^Department of Neurology, the First Affiliated Hospital of Zhengzhou University, Zhengzhou, Henan, China.; ^3^The Academy of Medical Sciences, Zhengzhou University, Zhengzhou, Henan, China.; ^4^Clinical Laboratory, the First Hospital of Yongnian District, Yongnian, Hebei, China.; ^5^Department of Pharmacy, the First Affiliated Hospital of Zhengzhou University, Zhengzhou, Henan, China.; ^6^Translational Medical Center, the First Affiliated Hospital of Zhengzhou University, Zhengzhou, Henan, China.; ^7^Brain Function and Disease Laboratory, Shantou University Medical College, Shantou, China.

**Keywords:** Alzheimer's disease, beta-amyloid, Neurodegeneration, Cathepsin family, Neuroinflammation

## Abstract

Alzheimer's disease (AD) is an intricate neurodegenerative disorder characterized by the accumulation of misfolded proteins, including beta-amyloid (Aβ) and tau, leading to cognitive decline. Despite decades of research, the precise mechanisms underlying its onset and progression remain elusive. Cathepsins are a family of lysosomal enzymes that play vital roles in cellular processes, including protein degradation and regulation of immune responses. Emerging evidence suggests that cathepsins may be involved in AD pathogenesis. Cathepsins can influence the activation of microglia and astrocytes, the resident immune cells in the brain. However, cathepsin dysfunction may lead to the accumulation of misfolded proteins, notably Aβ and tau. In addition, dysregulated cathepsin activity may induce an exaggerated immune response, promoting chronic inflammation and neuronal dysfunction in patients with AD. By unraveling the classification, functions, and roles of cathepsins in AD's pathogenesis, this review sheds light on their intricate involvement in this devastating disease. Targeting cathepsin activity could be a promising and novel approach for mitigating the pathological processes that contribute to AD, providing new avenues for its treatment and prevention.

## 1. INTRODUCTION

Alzheimer's disease (AD) ranks among the most prevalent neurodegenerative disorders [1]. AD is characterized by features, including memory loss, progressive decline in cognitive processing and reasoning, and a shift in personality and behavior, and has been established as the leading cause of dementia [2, 3]. Its pathological characteristics mainly manifest as the accumulation of extracellular beta-amyloid (Aβ) plaques in the brain parenchyma and intracellular neurofibrillary tangles (NFTs) composed of hyperphosphorylated Tau protein [4, 5]. Recent estimates suggest that over 55 million individuals worldwide are afflicted by AD, while the precise mechanisms governing its pathogenesis remain elusive [6]. Currently, there is no drug that can effectively reverse cognitive deterioration [7]. This devastating disease not only inflicts profound physical suffering upon patients but also imposes a substantial burden on families in terms of caregiving demands and emotional distress, further straining healthcare systems[8]. Therefore, identifying the molecular mechanisms of this disease and developing effective therapeutic drugs are necessary.

Cathepsins constitute a prevalent group of proteases in the endosome-lysosome system [9]. The etymology of “cathepsin” traces back to the Greek word for “digestion”, and its initial usage in the scientific literature was first introduced by *Willstätte* and *Bamann* in 1920 [10]. Since then, the structural features, distribution, substrate affinities, and clinical significance of the cathepsin family have been extensively explored and documented. These enzymes are present in various cell types throughout the body and perform crucial functions in a wide range of physiological processes, including digestion, blood coagulation, bone resorption, ion channel activity, innate immunity, complement activation, apoptosis, vesicular trafficking, autophagy, angiogenesis, proliferation, and metastasis [11].

Recent studies have highlighted the significant contributions of the cathepsin family to the pathogenesis of AD. For instance, cathepsin X (CTSX) participates in the regulation of microglial function, which affects chronic neuroinflammation in the brain and mediates neurodegenerative diseases, including AD [12]. Besides, an increase in the activity and expression levels of cathepsin B (CTSB) was found to correlate with increased amyloid plaque deposition [13]. Additionally, cathepsin D (CTSD), the predominant Aβ-degrading protease, has been implicated in the formation of NFTs [14]. Collectively, these findings underscore the potential of the cathepsin family as a promising therapeutic target in patients with AD. In this review, we systematically demonstrate the biological properties, structure, and functions of most cathepsins and present their regulatory roles in the progression of AD, offering valuable insights for the future treatment of AD.

**Figure 1.**
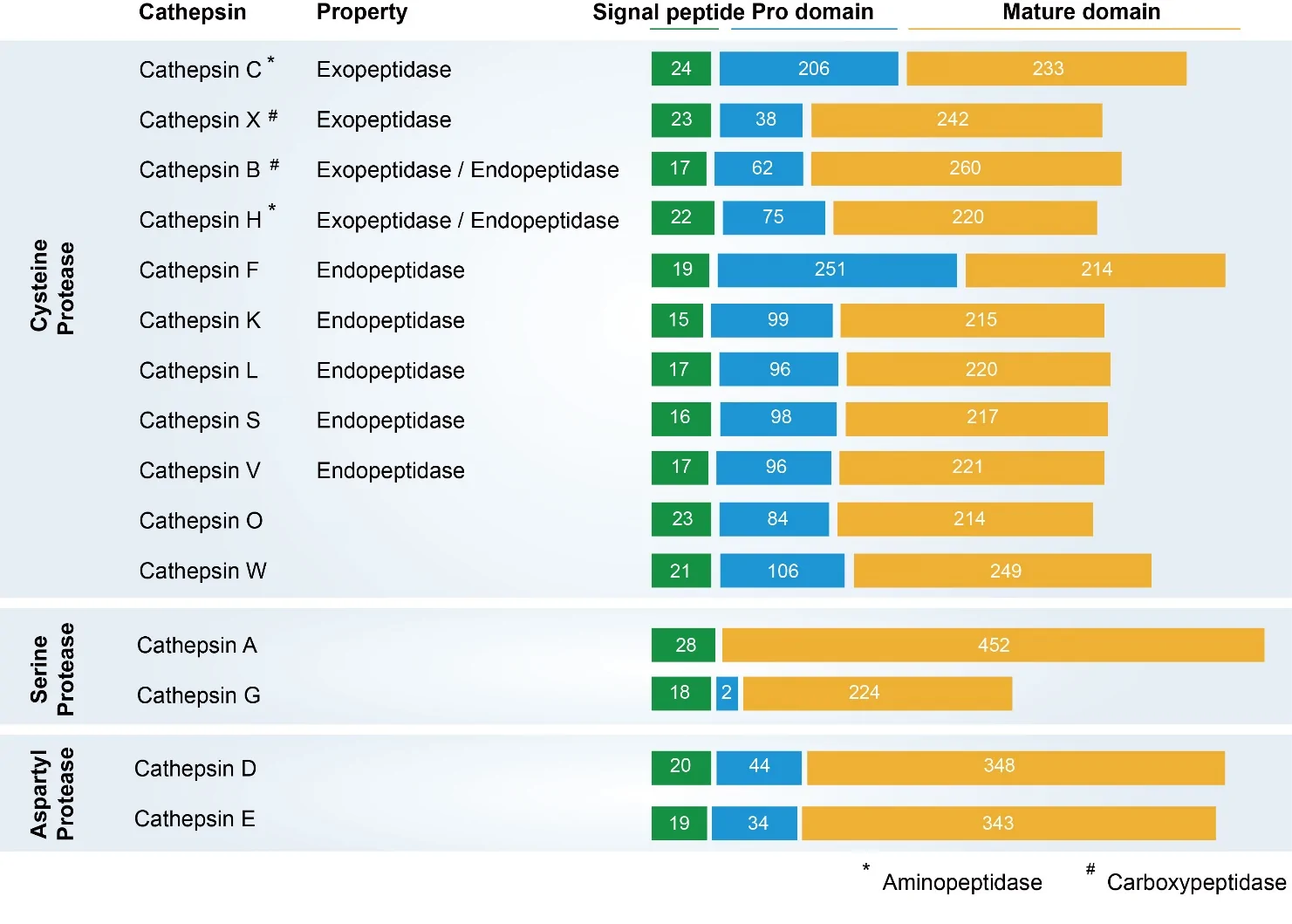
**Classification and Structural Schematic Representation of Human Cathepsins.** The human cathepsin family comprises eleven cysteine proteases, two serine proteases, and two aspartic proteases. Cysteine cathepsins can be further categorized into endopeptidases, exopeptidases, aminopeptidases, and carboxypeptidases based on substrate specificity. Cathepsins exhibit specific amino acid numbers and domain lengths, crucial features for understanding their functionality. A detailed representation of the functional sequences of cathepsins is provided, including their domain composition (signal peptide, prodomain, and mature domain), along with corresponding amino acid numbers.

**Table 1:** Classes, protease types, biological roles of cathepsins, and related diseases.

No.	Classes of cathepsins	Protease type	Mechanisms	Diseases	Reference
**1**	Cathepsin A	Serine protease	Processing of endogenous bioactive peptides; Inhibit autophagy	Muscular dystrophy; Galactosialidosis	[39]
**2**	Cathepsin B	Cysteine protease	Promotes amyloid plaque; Matrix degradation and cell invasion; Enables virus entry into the cells	Alzheimer's disease; Cancer	[54]
**3**	Cathepsin C	Cysteine protease	Inflammation; Catalyzes the excision of dipeptides from the N-terminus of protein and peptide substrates	Papillon-Lefevre disease; Keratosis Periodontitis	[35]
**4**	Cathepsin D	Aspartyl protease	Mitogen and promotes invasiveness; Cleaves ECM proteins	Breast cancer; Possibly Alzheimer disease; Neuronal ceroid lipofuscinosis (NCL)	[38]
**5**	Cathepsin E	Aspartyl protease	Antigen processing via the MHC class II pathway	Atopic dermatitis	[51]
**6**	Cathepsin F	Cysteine protease	Contains five potential N-glycosylation sites, and it may be targeted to the endosomal/lysosomal compartment via the mannose 6-phosphate receptor pathway	Type B Kufs disease	[18, 56]
**7**	Cathepsin G	Serine protease	Plays an important role in eliminating intracellular pathogens and breaking down tissues at inflammatory sites, as well as in anti-inflammatory response	Tuberculosis; Rheumatoid arthritis; Coronary artery disease; Periodontitis; Ischemic reperfusion injury	[40]
**8**	Cathepsin H	Cysteine protease	Endopeptidase activity	Prostate tumors Severe myopia; Diabetes mellitus type 1	[17]
**9**	Cathepsin K	Cysteine protease	Cleaves ECM protein collagen; Secretion by osteoclasts in bone resorption	Osteoporosis; Arthritis; Atherosclerosis; Obesity; Schizophrenia; Cancer metastasis	[42]
**10**	Cathepsin L	Cysteine protease	Matrix degradation and cell invasion; Enables virus entry into the cells	Cancer; Gingival overgrowth	[44, 45]
**11**	Cathepsin O	Cysteine protease	Collagenolysis; Elastinolysis; Osteoclastic bone resorption	Cardiovascular disease	[32]
**12**	Cathepsin S	Cysteine protease	Antigen presentation; Remodeling of connective tissue and basement membranes	Type IV astrocytomas (glioblastoma multiforme); Atherosclerosis	[50]
**13**	Cathepsin V	Cysteine protease	Production of enkephalin and neuropeptide Y	Keratoconus	[36]
**14**	Cathepsin W	Cysteine protease	Cell-mediated cytotoxicity	Inflammatory bowel disease; Autoimmune gastritis	[33]
**15**	Cathepsin Z	Cysteine protease	Protein degradation	Cancer; Inflammation	[34]

## 2. BIOLOGICAL FEATURES OF CATHEPSINS

### 2.1 Classification and synthesis of cathepsins

**Figure 2.**
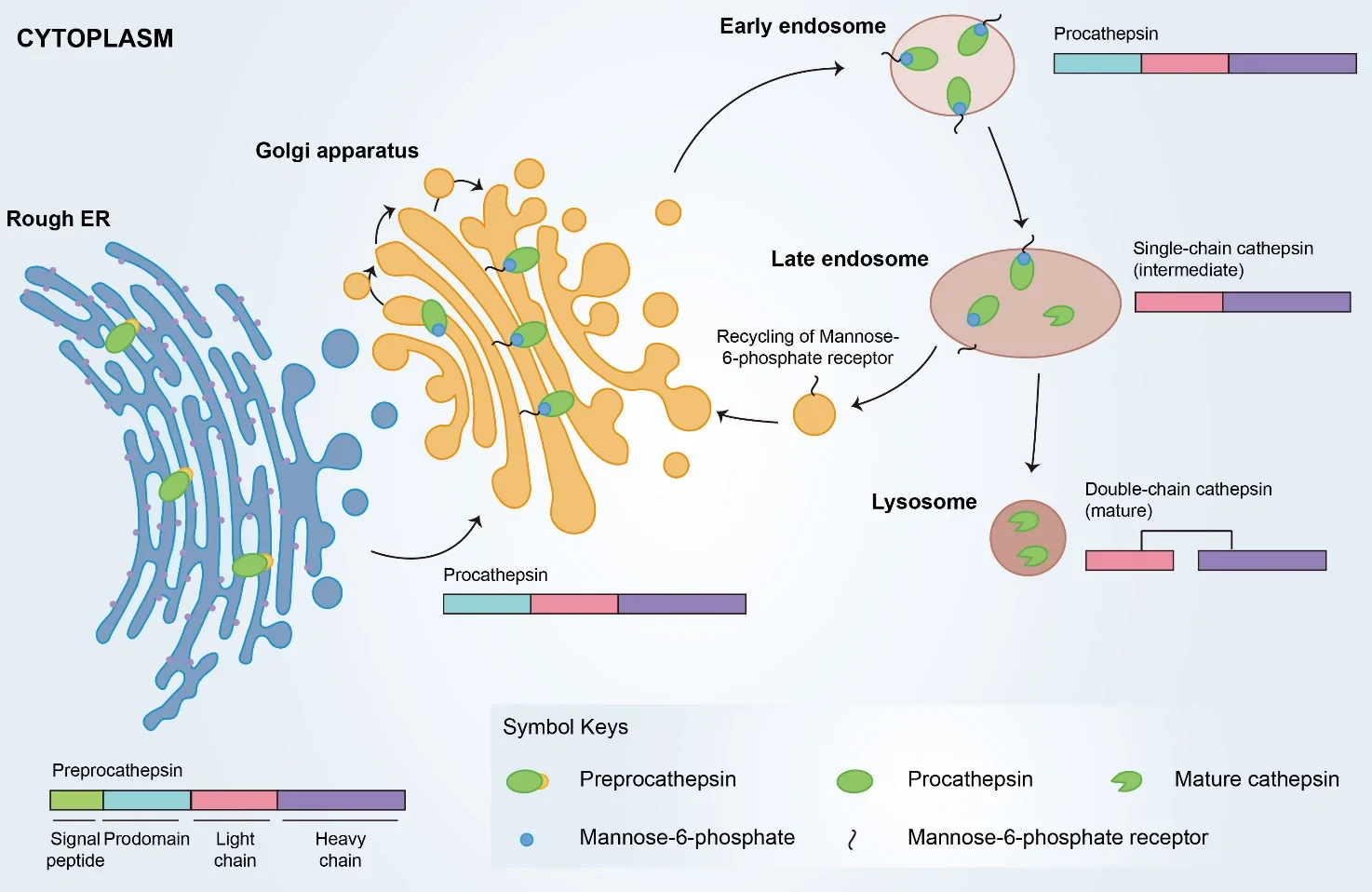
**Schematic illustration showing the maturation process of lysosomal cathepsins**. Cathepsins are synthesized as preprocathepsin with an N-terminal signal peptide that targets the protein to the lumen of the rough endoplasmic reticulum (RER), where it undergoes signal peptide hydrolysis and glycosylation. Following this, they progress to the Golgi apparatus and early endosome for further maturation, culminating in the formation of mannose-6 phosphate (Man-6-P) proteins. In the late endosomes, the pH decreases, leading to the separation of procathepsins from the Man-6-P receptor, resulting in the release of proteolytically active single-chain intermediate cathepsins. Within the lysosome, the single-chain protein undergoes further processing through autocatalysis, ultimately forming a mature two-chain structure consisting of an N-terminal light chain and a C-terminal heavy chain.

In accordance with variations in their catalytic sites, categorized into distinct families, as presented in Fig. 1 and Table 1. In humans, the cathepsin family includes eleven cysteine proteases (cathepsin B, C, F, H, K, L, O, S, V, W, and X/Z), two serine proteases (cathepsin A and G), and two aspartic proteases (cathepsin D and E) [15]. Based on their substrate specificity, cysteine cathepsins can be further classified into peptide endonucleases, peptide endlylases (peptide exonucleases), aminopeptidases, and carboxypeptidases. Peptide endonucleases include cathepsin B, F, H, K, L, S, and V; peptide endlylases include cathepsin B, C, H, and X; aminopeptidases include cathepsins C and H; and carboxypeptidases include cathepsins B and X. Notably, cathepsins B and H exhibit dual functions as both endopeptidases and exopeptidases [16, 17]. Virtually all cathepsins are synthesized in a precursor form within the ribosome, comprising a signal peptide, a precursor peptide, and a catalytic domain. These precursors are subsequently transported to the endoplasmic reticulum via transferrin, where they undergo signal peptide hydrolysis and glycosylation. Next, they progress to the Golgi apparatus for further maturation, culminating in the formation of mannose-6 phosphate (Man-6-P) proteins. These mature proteins are then recognized by mannose 6-phosphate receptors on lysosomes and subsequently directed to the lysosomal system[18], where they ultimately become fully functional mature cathepsins [19] (Fig. 2).

### 2.2 Three-dimensional (3D) structure of cathepsins

Cysteine cathepsins belong to the papain family and are characterized by their typical papain-like fold structures. It is composed of three-dimensional pocket-like structures formed by histidine (His), asparagine (Asn), and cysteine (Cys) residues, including signal peptides, precursor peptides, and catalytic domains containing mature active centers. In humans, the amino acid sequences of cathepsins are similar to those of papain, featuring a conserved active site consisting of the Cys25, His159, and Asn175 residues [20]. Over the years, extensive structural studies have unveiled the intricate interactions of these residues with both substrates and inhibitors, thereby offering invaluable insights into the substrate specificity and catalytic mechanisms of cathepsins. The three-dimensional structures of cathepsins are highly conserved and comprise almost the same L domain and R domain. The L domain contains three α-helices, of which the longest α-helix (central helix) consists of approximately 40 amino acid residues, while its N- terminus contains the conserved site Cys25. The R domain has a barrel-shaped structure and is mainly composed of 5-6 β-chains that contain the conserved His159 and Asn175 sites. Notably, in the majority of cathepsins, the L domain features at least two disulfide bonds, enhancing the stability of this domain. In contrast, the R domain contains a single disulfide bond situated between the L domain and the R domain, forming a narrow V-shaped cleft that serves as the catalytic active site [21-23].

### 2.3 Substrate Specificity of Cathepsins

The three-dimensional (3D) structures of cathepsins play a critical role in determining their substrate specificity. These structures dictate how cathepsins recognize and process specific target molecules. Cysteine cathepsins, including cathepsin B, C, L, and S, possess an active site containing a nucleophilic cysteine residue essential for catalysis [24]. The surrounding amino acid residues form a binding pocket specific for particular substrates. These pockets often contain aromatic amino acids such as phenylalanine, tryptophan, or tyrosine [25]. Consequently, cysteine cathepsins exhibit a preference for cleaving peptide bonds located C-terminal to aromatic residues [26]. Aspartic cathepsins, including cathepsin D and E, are characterized by the presence of two aspartic acid residues at their active site. These residues form a catalytic dyad with a water molecule, enabling activity across a broad pH range, particularly in acidic environments [9]. Aspartic cathepsins typically target peptide bonds adjacent to acidic or basic amino acid residues. Serine cathepsins, while less common within lysosomes, are found in other cellular compartments like the pancreas (e.g., trypsin). They are characterized by a catalytic triad consisting of a serine residue, along with histidine and aspartate residues [27]. These proteases are versatile, capable of cleaving a wide array of peptide bonds, particularly those located after neutral or basic amino acids [24].

The three-dimensional (3D) structure of cathepsins is critical for their function. Specific structural motifs within these enzymes govern their ability to recognize and process specific substrates. Understanding these structural nuances is fundamental to deciphering the roles of cathepsins in various biological processes and for the development of novel therapeutic strategies. Extensive research has already elucidated many of these structural features, highlighting the importance of further investigation into their functional significance. This detailed knowledge of cathepsin structures lays the groundwork for exploring their diverse roles in health and disease, which will be discussed in the following section.

### 2.4 Interaction of Cathepsin Domains with Inhibitors

Domains within the structure of cathepsin proteases play a crucial role in inhibitor binding. These interactions modulate enzyme activity by forming stable complexes. The specificity of these interactions is vital for the development of targeted therapies. It allows for the regulation of cathepsin activity with minimal off-target effects. Cysteine cathepsins, including cathepsin B, C, L, and S, possess an active site cysteine residue. This residue serves as the primary target for many inhibitors [28]. The subsites adjacent to the active site form a binding cleft that accommodates the P1 residue of the inhibitor, facilitating specific targeting [29]. E-64 exemplifies a widely recognized cysteine protease inhibitor. It targets cathepsin B by binding it to its active site, thereby inhibiting its proteolytic activity. X-ray crystallography has elucidated the structural basis for this interaction, providing valuable insights into the molecular recognition process [30]. Aspartic cathepsins, including cathepsin D and E, feature two aspartic acid residues in their active site. These residues are targeted by inhibitors such as pepstatin [31]. The binding of these inhibitors to the active site disrupts the catalytic mechanism of the enzyme.

Understanding the structural domains of cathepsins and their interactions with inhibitors forms the foundation for developing selective and potent therapeutic agents. Techniques in structural biology, particularly X-ray crystallography, have been instrumental in elucidating the molecular details of these interactions. This knowledge is critical for the rational design of inhibitors with targeted activity.

The structural complexity of the cathepsin family members underscores their multifaceted functions and regulatory mechanisms. Advances in structural biology techniques in recent years have provided invaluable insights into their activation, substrate binding, and regulatory processes. These findings not only enhance our understanding of the physiological roles of cathepsins but also pave the way for the development of novel therapeutic interventions targeting these proteases in disease contexts.

## 3. BIOLOGICAL FUNCTIONS OF CATHEPSINS

The cathepsin family comprises a remarkably diverse group of proteases, each of which contributes to a wide spectrum of physiological functions critical for maintaining overall health and cellular homeostasis [32-36].

### 3.1 Roles of Cathepsins in Protein Degradation

Cathepsins play a critical role in lysosomal protein degradation. These enzymes recognize and cleave target proteins, facilitating a vital cellular process. This process, known as lysosomal proteolysis, allows for the turnover of cellular components and the recycling of amino acids[37]. Cysteine cathepsins (B, L, S, etc.), aspartic cathepsins (D, E) [38], and serine cathepsins (A, G) [39, 40] participate in the proteolytic processing and clearance of cellular proteins, thereby regulating protein turnover and maintaining cellular health [40, 41]. Besides, cathepsin K, a prominent member of a family known for its role in bone remodeling [42], is reportedly involved in the degradation of collagen and elastin in the extracellular matrix, contributing to tissue repair and bone resorption [43-45]. Recent research suggests a broader role for cathepsins (D, L) in endoplasmic reticulum-associated degradation (ERAD), highlighting their multifaceted roles within the cellular machinery [46, 47].

### 3.2 Involvement of Cathepsins in Cellular Processes

Beyond protein degradation, cathepsins interact with multiple cellular processes, including autophagy. Yu-Qin Di *et al.* found that the expression level and autophagy-mediated maturation of cathepsin D in tissues determine its roles in apoptosis and cell proliferation. This, in turn, determines the cell fates of tissues during lepidopteran metamorphosis [48]. Current evidence also suggests that cathepsin B modulates autophagy processes in adipocytes [49]. Moreover, certain cathepsins, most notably cathepsin L and cathepsin S, play indispensable roles in antigen processing within antigen-presenting cells [50, 51]. They cleave antigens within endosomes and lysosomes to generate peptide fragments for presentation by major histocompatibility complex (MHC) molecules, facilitating immune recognition and response [32].

**Figure 3.**
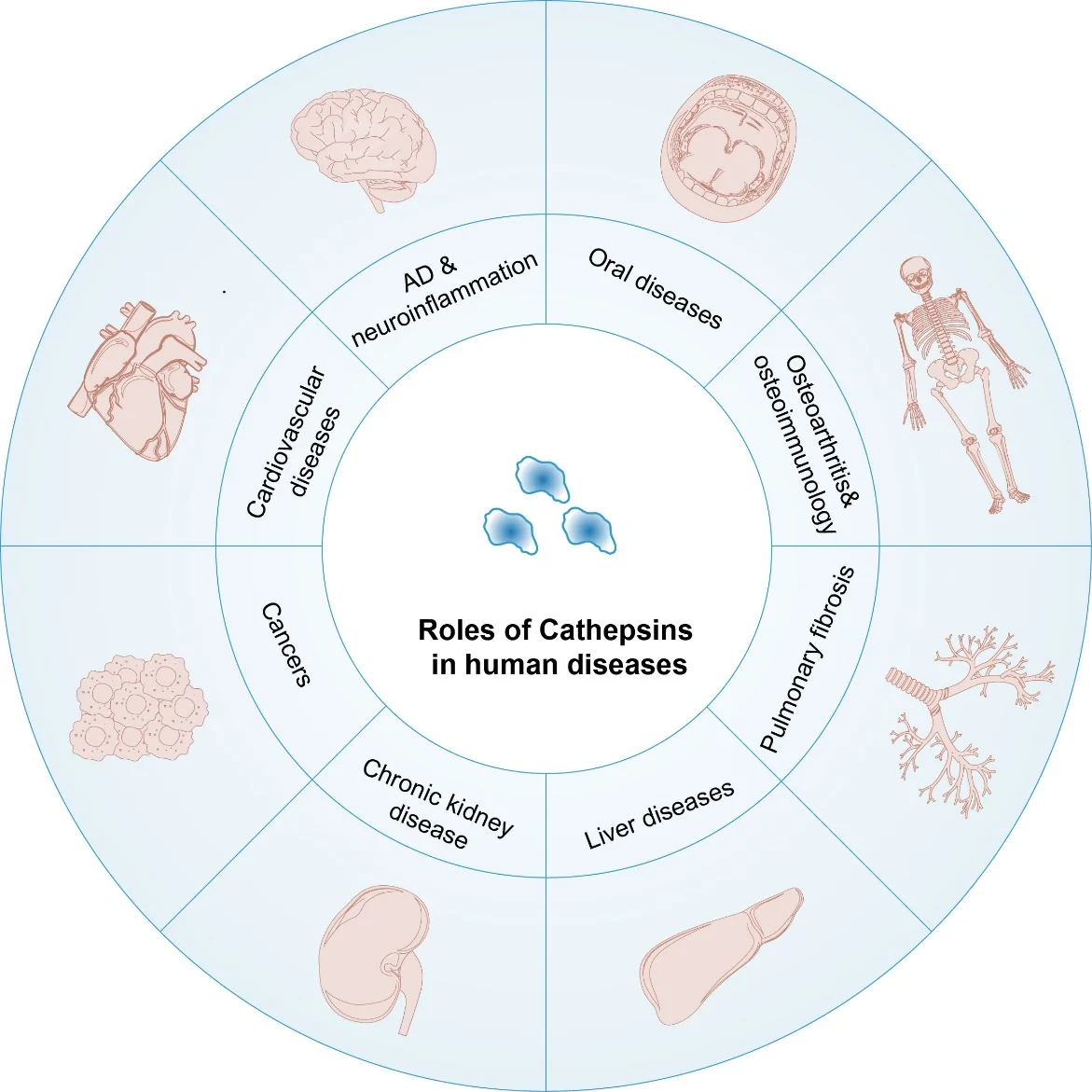
**Cathepsins in human diseases**. This schematic overview depicts various medical conditions where abnormal cathepsin activity or expression has been documented or linked to disease progression, including oral disease, osteoarthritis and osteoimmunology, cardiovascular diseases, pulmonary fibrosis, chronic kidney disease, cancers, liver diseases, AD and neuroinflammation, and so on.

### 3.3 Cathepsin Involvement in Disease Pathogenesis

Cathepsins have been implicated in various human diseases and processes, including oral disease, osteoarthritis and osteoimmunology, cardiovascular diseases, pulmonary fibrosis, chronic kidney disease, cancers, liver diseases, AD and neuroinflammation, and others (Fig. 3). In addition, various cathepsins are crucial in maintaining tissue homeostasis. Cathepsin B, for instance, exerts its influence within the brain, regulating neurite outgrowth and synaptic plasticity [52]. Meanwhile, cathepsin D plays a vital role in liver function, participating in the breakdown of cellular debris and ensuring proper organ health [53]. Notably, cathepsins are also involved in cell migration and invasion processes. Cathepsin B, L, and S, for example, actively facilitate cancer metastasis by promoting the degradation of the extracellular matrix, thereby enhancing the invasive potential of cancer cells [54]. Furthermore, cathepsins have been implicated in the intricate regulation of apoptosis. Cathepsin D plays a role in apoptotic processes by cleaving apoptotic substrates, contributing to the regulation of programmed cell death [55, 56]. Importantly, recent studies have revealed a growing association between the cathepsin family and neurological functions. In addition to their roles in neuronal development and synaptic plasticity, cathepsins, particularly cathepsin D, have been linked to neurodegenerative diseases such as AD and Parkinson's disease (PD), in which they participate in the processing of pathological proteins [14, 38].

In conclusion, the cathepsin family of proteases has emerged as a versatile and indispensable group of proteins with diverse physiological functions. Their contributions span from protein degradation and immune responses to tissue remodeling and cellular regulation, signifying their paramount importance in maintaining overall health and cellular balance.

## 4. CATHEPSINS IN AD PATHOLOGY

Over the years, multiple hypotheses have been proposed to explain the pathogenesis of AD, including the amyloid cascade hypothesis [57], the Tau propagation hypothesis [58], the cholinergic hypothesis [59], the calcium homeostasis hypothesis [60], the neuroinflammation hypothesis [61], the mitochondrial cascade hypothesis [62], and the metal ion hypothesis [63], to name a few.

**Figure 4.**
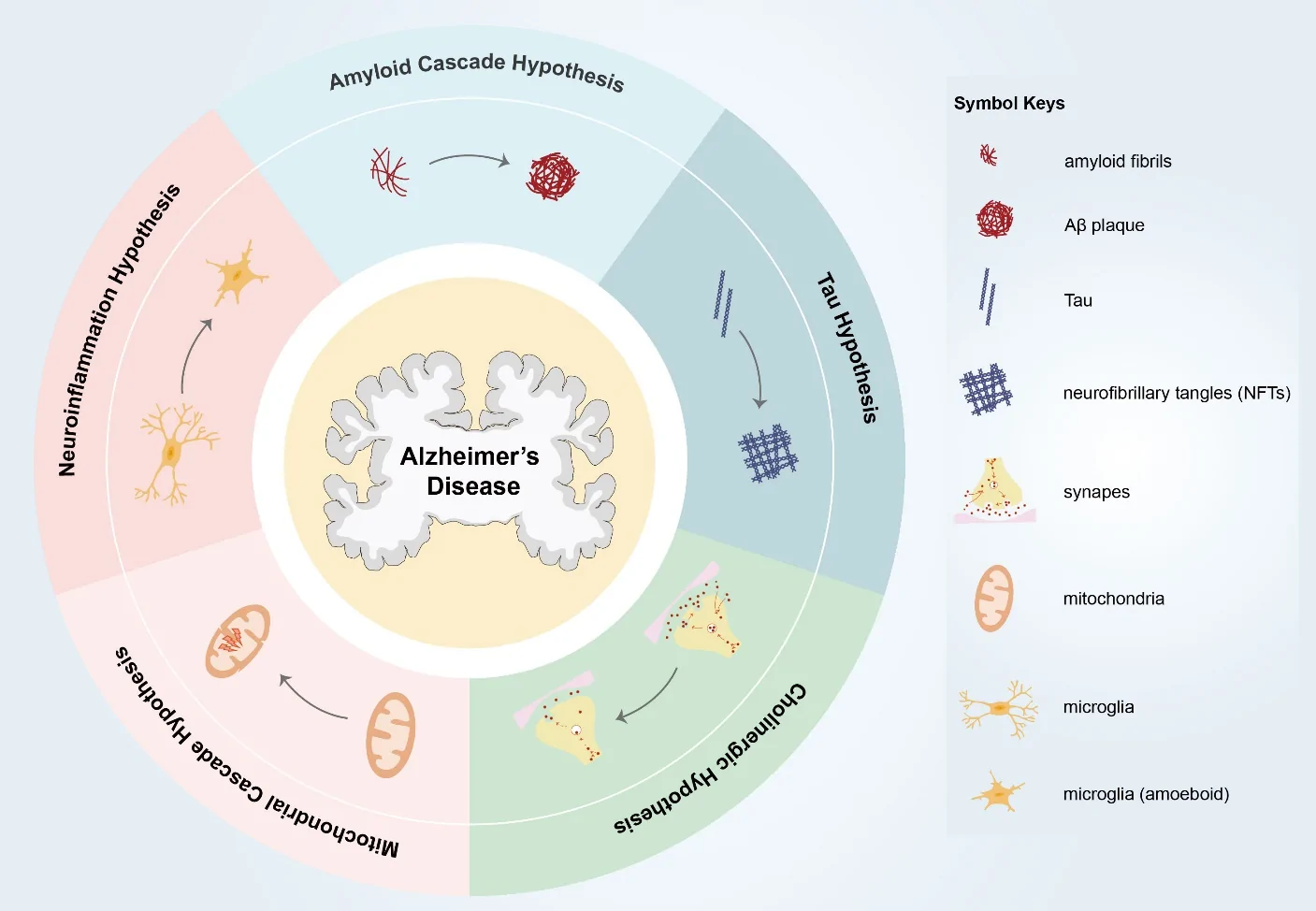
**Schematic illustration showing the five main hypotheses of AD**. The five main hypotheses for the onset and progression of AD, including the amyloid cascade hypothesis, the tau hypothesis, the neuroinflammation hypothesis, the mitochondrial cascade hypothesis, and the cholinergic hypothesis, are presented.

As shown in Figure 4, we detailed the five main hypotheses. At the core of the amyloid cascade hypothesis are Aβ peptides. Aβ is a proteolytic fragment of APP composed of 39-43 amino acids and is considered the molecular driver of AD pathogenesis and progression [64]. The most abundant forms of Aβ are Aβ40 and Aβ42, with Aβ42 possessing a more potent neurotoxicity [65]. Aβ aggregates are thought to exert neurotoxic effects through several mechanisms. They can trigger inflammatory responses and oxidative stress and disrupt synaptic function, leading to neuronal injury and death. This cascade of events ultimately results in the progressive cognitive impairment characteristic of AD [66]. Besides, one crucial aspect of the Aβ hyp289othesis is the concept of a "cascade effect", which suggests that Aβ accumulation acts as a trigger for further pathological events, including tau protein hyperphosphorylation and the formation of NFTs [67]. Tau is a microtubule-associated protein primarily found in neurons. Its primary role involves stabilizing microtubules, crucial for preserving the structural integrity and facilitating the transportation of neuronal cells [68]. However, in AD, tau becomes abnormally phosphorylated and aggregates, which interfere with microtubule stability, disrupting crucial intracellular transport processes and impairing synaptic function. These disruptions lead to neuronal dysfunction and, ultimately, cell death. The neuroinflammation hypothesis is a compelling theory in AD research that has gained prominence in recent years [69]. Emerging research has revealed that the brain possesses its own immune system, primarily comprised of microglia and astrocytes. In AD, there is sustained and chronic activation of microglia and astrocytes, leading to an inflammatory state within the brain [70]. Interestingly, activated microglia and astrocytes release inflammatory mediators, including pro-inflammatory cytokines, chemokines, and reactive oxygen species (ROS), further exacerbating neuroinflammation and contributing to neurodegeneration, synaptic dysfunction, and cognitive decline in AD patients.

**Figure 5.**
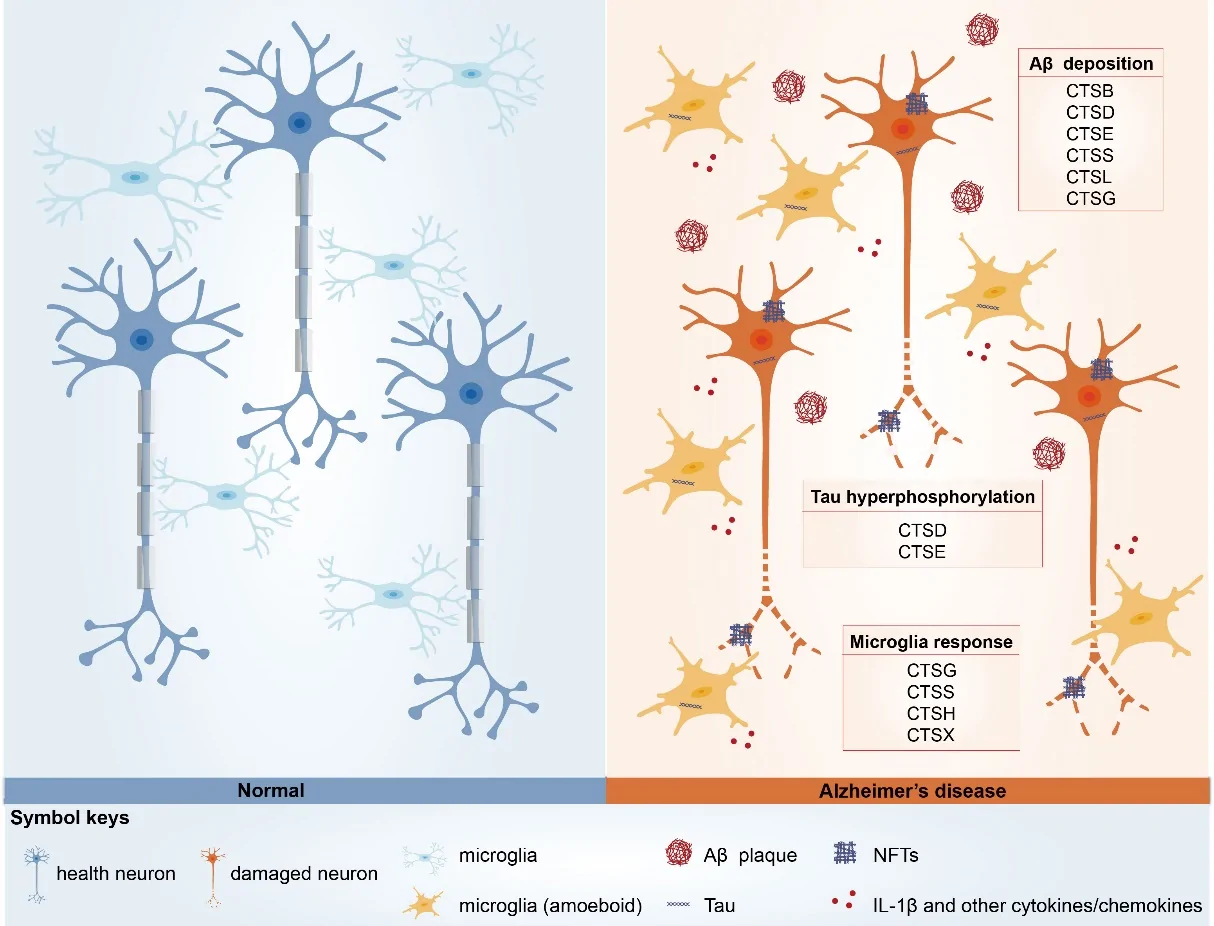
**Schematic illustration showing the role of the cathepsins in the progression of AD. (Left)** In a healthy brain, microglia exhibit a ramified morphology, enveloping neurons and providing protective functions by facilitating Aβ clearance through phagocytosis to restore tissue homeostasis. **(Right)** Conversely, in the AD-afflicted brain, microglia are consistently activated and display an amoeboid morphology, contributing to neuroinflammation. Furthermore, the deposition of Aβ plaques and aggregation of hyperphosphorylated tau in neurofibrillary tangles (NFTs) are also observed in damaged neurons. The cathepsin family plays a crucial role in driving these pathological changes. CTSB, CTSE, CTSS, CTSL, and CTSG are involved in regulating Aβ deposition, while CTSD and CTSE are pivotal in the hyperphosphorylation of tau. Additionally, CTSG, CTSH, CTSS, and CTSX take part in the immune response of microglia.

The cathepsin family has emerged as a critical player in the progression of AD. As shown in Figure 5, their involvement in Aβ deposition, Tau hyperphosphorylation, and microglia response underscores the relevance of these proteins in AD pathogenesis. We demonstrate the biological properties, structure, and function of most cathepsins and present their regulatory function in the progression of AD. Understanding the complex interplay between cathepsins and AD pathology holds promise for developing novel therapeutic interventions aimed at mitigating the progression of this devastating neurodegenerative disorder.

### 4.1 Cathepsin B

One of the most abundant lysosomal cathepsins in the human brain is cathepsin B (CTSB), which is a cysteine cathepsin [71]. The crystal structure of CTSB, which was isolated from the human liver by R. Huber *et al.*, was unknown until the 1990s [72]. The *CTSB* gene, which has 13 exons and 11 introns and is approximately 27 kb in length, is located in the p22 region of chromosome 8 [73]. The molecular weight of CTSB, which encodes a precursor of 339 amino acids, is approximately 30 kDa and is composed of a heavy chain of 26 kDa and a light chain of 5 kDa. The structure of CTSB is spherical, and the active site and substrate are located at the interface between the two subunits. Cleavage of the peptide bond is accomplished by the interaction of a cysteine residue on the light chain (Cys29) and a histidine residue on the heavy chain (His199). In the case of active sites, the sulfhydryl and imidazolyl groups of Cys29 and His199 can form ion pairs in the pH range of 4.0 to 8.5, and the cleavage of substrate peptide bonds is mediated by nucleophilic attack on Cys29 [72]. In addition to being an endogenous peptidase that cleaves the internal peptide bond to form a large hydrophobic bond in the substrate, CTSB is also a peptidyl dipeptidase, whose internal structure can block the end of the active site gap and recognize positively charged imidazole groups on heavy chains [74]. CTSB is located mainly in subnuclear endosomes and lysosomes, contributing to the exchange of intracellular and extracellular proteins [75]. It plays vital roles in phagocytosis, autophagy, tumor cell growth and proliferation, angiogenesis, invasion, and metastasis [76]. In addition, CTSB exhibits selective high expression in hippocampal neurons and in the cerebral cortex of healthy mice [77].

The role of CTSB in the pathogenesis of AD has been increasingly documented in several studies. It has been shown that AD patients have higher CTSB serum levels, which is positively associated with cognitive dysfunction [78]. In a study conducted by Kindy MS *et al.*, AD model mice expressing hAβPP in SAD patients were first constructed, with *CTSB*-knockout mice exhibiting significantly improved memory during the Morris’s water maze (MWM) test and reduced Aβ deposition in contrast to control mice. Moreover, the peptide levels of Aβ1-40, Aβ1-42, pGlu-Aβ3-40 and pGlu-Aβ3-42 were decreased in the brains of the CTSB-knockout mice [79]. Wu *et al.* further suggested that *Porphyromonas gingivalis* lipopolysaccharide (PgLPS) induces Aβ deposition, as well as, learning and memory deficits. Interestingly, these deficits were not observed in *CTSB*-KO mice. This finding aligns with the observation that periodontitis is associated with cognitive decline in Alzheimer's patients. In a separate study, researchers showed that decreasing CTSB levels improved learning and memory function in mice with a periodontitis-induced AD model [80, 81]. However, Embury *et al.* reported conflicting results, demonstrating that CTSB overexpression promoted Aβ degradation, inhibited Aβ deposition in neurons, and restored memory and learning function in APP/PS1 mice [82].

The role of CTSB in AD appears multifaceted and may be influenced by several factors. These include the experimental models used, the specific CTSB functions assessed, and the disease stages investigated. CTSB exhibits a dual role in Aβ metabolism, encompassing both the generation and degradation of Aβ peptides. Some studies suggest that CTSB can cleave the Amyloid Precursor Protein (APP) at a site distinct from the β- and γ-secretases, potentially contributing to Aβ generation [83]. Conversely, CTSB is also recognized for its ability to degrade Aβ peptides within the lysosomal compartment [84]. Effective Aβ degradation by CTSB is crucial for maintaining Aβ homeostasis and preventing their accumulation into toxic aggregates. The dual role of CTSB in AD is likely context-dependent. The concentration of CTSB and its substrates can influence whether it promotes Aβ generation or degradation. An excess of CTSB or impaired lysosomal function might shift the balance towards Aβ production. Additionally, the acidic environment of the lysosome, where CTSB is most active, can affect the specificity of its proteolytic actions. Any alteration in lysosomal pH or biogenesis could impact CTSB's ability to degrade Aβ. The activity of CTSB itself can also influence its role in Aβ metabolism. Endogenous inhibitors, such as stefin A, and activators regulate CTSB activity [85]. An imbalance in these regulators could affect CTSB's role in Aβ metabolism. Furthermore, post-translational modifications like glycosylation or phosphorylation could alter CTSB's activity or its access to substrates, thereby influencing Aβ metabolism [86]. In summary, CTSB's role in Aβ metabolism is complex and may be a key factor in AD development and progression. Further research is warranted to fully elucidate the mechanisms underlying CTSB's dual function and to exploit this knowledge for therapeutic benefit.

### 4.2 Cathepsin D

Cathepsin D (CTSD) is an aspartate lysosomal peptide endonuclease discovered by Westley *et al.* in 1979 and belongs to the family of aspartate cathepsins. The human *CTSD* gene is located at chromosomal region 11 p15.5, consists of 9 exons and encodes a protein of 412 amino acid residues. The mature CTSD consists of a 34kDa heavy chain and a 14kDa light chain [9]. CTSD matures gradually through the secretory pathway and is an inactive precursor of CTSD until it reaches the lysosome.

The 52 kDa precursor of cathepsin D is hydrolyzed and processed into a 48 kDa intermediate form in the lysosome after passing through the endoplasmic reticulum, where it is then further processed into mature cathepsin. An acidic environment is essential for the proteolytic processing and maturation of CTSD. The mature CTSD is composed of two domains, each of which provides an aspartic acid group to the catalytic site. The active site is vulnerable to dehydrogenation because of these two aspartic acid groups, which increase cathepsin activity in acidic environments [87-89]. CTSD is expressed in most cells. Additionally, the brain typically exhibits elevated CTSD expression levels [90].

According to the research conducted by *Josina Bunk et al.*, CTSD is involved in the lysosomal circulation and degradation of a wide range of substrates, and the occurrence and progression of several neurodegenerative diseases, including AD, were linked to *CTSD* gene mutations [89]. CTSD has emerged as a key player in the proteolytic clearance of Aβ, contributing to the maintenance of cellular proteostasis [91]. Besides, numerous CTSD cutting sites have been documented on Tau proteins, as CTSD plays a significant role in the degradation of nerve fiber junctions [92]. Chai Y L *et al.* conducted a controlled clinical trial and assessed the serum CTSD concentration in 34 patients with AD dementia, 40 patients with cognitive dysfunction but no dementia, and 35 patients without cognitive dysfunction. Patients with AD-related dementia presented with higher serum CTSD concentrations than patients in the other two groups [93]. Another study by the same research group revealed increased immune activity of CTSD in the cortex of AD patients, and CTSD was partially colocalized with neurons containing NFTs, highlighting the positive correlation between CTSD and the formation of NFTs [94]. In addition, CTSD was found to colocalize with Aβ plaques [65]. A study by Suire C. N. *et al.* revealed that CTSD was the main protease involved in the degradation of Aβ and affected the ratio of Aβ42/Aβ40 through the degradation of Aβ42 and Aβ40 [95]. In summary, CTSD emerges as a critical lysosomal enzyme with significant implications for AD pathology. CTSD's role in degrading Aβ and Tau proteins, coupled with its altered expression observed in AD patients, makes it a promising therapeutic target for AD.

### 4.3 Cathepsin H

Cathepsin H (CTSH) is also a member of the cathepsin family and a cysteine cathepsin [96], that was first isolated from the liver of a mouse. The *CTSH* gene is located in the q24-q25 region of chromosome 15. Mature CTSH has a molecular weight of approximately 28 kDa and is composed of a heavy chain with a molecular weight of 22 kDa and a light chain of 5-6 kDa. Similar to CTSB, CTSH also exhibits both exopeptidase and endopeptidase activities [97, 98]. CTSH is a cysteine protease commonly expressed in tissues or cells that is involved in cell apoptosis and plays a role in the development of cancer [99]. Previous meta-analyses have shown that *CTSH* is a new AD-related gene [100]. Through functional genomics analysis, Li Y, et al. demonstrated that elevated CTSH expression is a risk factor for AD. They also examined how the functional *CTSH* variant rs22889702, which is linked to genetic functions, has a protective effect on AD and was confirmed in the Chinese Han population [101]. Microglia play a significant role in the pathogenesis of AD, and improper activation of microglia induces neuroinflammation and exacerbates the progression of AD [69, 102, 103]. Studies have shown that CTSH is crucial during inflammatory response and tumor metastasis [15, 104]. After systemic injection of LPS, Fan K et al. discovered that CTSH levels in microglia were increased, and CTSH could increase the release of the inflammatory cytokines IL-1β and interferon-γ. However, the administration of CTSH inhibitory antibodies could decrease the protein levels of IL-1β and interferon-γ [105]. Taken together, these findings indicate that CTSH is involved in the neuroinflammatory response and is related to AD progression.

In summary, CTSH plays a multifaceted role in AD. Its expression in microglia and its ability to modulate cytokine release can significantly impact neuroinflammation. This dual role, as both a risk factor and a participant in the neuroinflammatory process, highlights CTSH's potential as a therapeutic target for AD.

### 4.4 Cathepsin S

Another member of the cathepsin family, cathepsin S (CTSS), is also a cysteine cathepsin. CTSS was first isolated from calf lymph nodes by Turnsek *et al.* in 1975, and it was not until 1992 that CTSS was first extracted from human alveolar macrophages [106, 107]. The human *CTSS* gene is located at chromosomal region 1q21 and includes 5 exons and 4 introns. Like other cathepsins, CTSS exist as zymogen precursors before they reach the lysosomes. The CTSS protein is encoded by 331 amino acids and can be divided into three different domains: the mature domain (which contains 217 amino acids), the propeptide domain (98 amino acids), and the signal domain (16 amino acids) [107-109]. The crystal structure of the mature CTSS is similar to that of the papain family, which contains a single-stranded protein of 225 amino acids with a relative molecular mass of approximately 24.8 kDa. The three-dimensional space includes two domains, the L domain, and the R domain. The L domain consists of three α helical structures and a hydrophobic pocket, while the R domain is composed of an antiparallel β -fold with one hydrophobic core embedded and two α -helices on the side [110]. In contrast to other cathepsins, CTSS activity is not dependent on the acidic environment. It can retain its activity in neutral and moderately alkaline pH. CTSS is expressed mainly on macrophages and antigen-presenting cells in the spleen and on lymphocytes. It primarily contributes to immune control and antigen presentation. Additionally, it has been demonstrated that CTSS is vital in mediating tumor invasion and angiogenesis in the inflammatory microenvironment of tumors and other inflammatory disease states [111, 112].

To compare the expression levels of lysosomal enzymes at various stages of AD, Morena F *et al.* collected blood samples from patients with mild AD, severe AD, or mild cognitive impairment (MCI). They selected glycohydrolase and cathepsin as the two lysosomal enzyme types most closely associated with AD based on their prior research. The results revealed that CTSS scores were greater in patients with AD than in those with MCI, which indicates that CTSS may be a potential target for the diagnosis of AD [113]. In addition, Schechter I *et al.* noted that CTSS can decompose amyloid precursor protein (APP) at a rate 1170 times faster than that of the β-secreting enzyme [114]. Although CTSS expression is low in normal brain tissue, it is increased in AD. Collectively, the above studies overlap in their assertion that CTSS plays a vital role in the development of AD. In conclusion, the collective body of research suggests that CTSS plays an intricate role in the pathogenesis of AD, potentially through its effects on APP processing and its contribution to neuroinflammation.

### 4.5 Cathepsin E

Cathepsin E is an aspartate cathepsin that was first discovered in the pancreas in 1996. The human *CTSE* gene, which has nine exons, is located in the chromosomal region 11q31-32. Mature CTSE is approximately 82 kDa in size and encodes a precursor consisting of 438 amino acid residues [115]. It has been established that an acidic environment is also necessary for its activity. Compared with CTSD, the activity of CTSE is less dependent on pH [116]. Cathepsin E is expressed in various tissues and organs. In humans, CTSE is mainly distributed in the immune system, gastrointestinal system, lymphoid tissues, and erythrocytes [117]. CTSE is generally not detectable under normal conditions and can only be detected in the pathological state [118]. Several studies have indicated that CTSE plays an important role in neurodegenerative diseases and is upregulated in AD patients [119].

Zhen Xie *et al.* uncovered that both AD patients and AD model mice exhibited higher CTSE expression [120]. Knockout of CTSE in AD model mice improved memory and learning behaviors significantly by inhibiting the expression of β-secretase APP and decreasing the deposition of Aβ. Moreover, CTSE knockout significantly reduced the phosphorylation of Tau protein on several residues, including 202 and 396. Besides, CTSE participates in the inflammatory response of microglia and mediates the occurrence of AD by affecting the soluble channel sTRAIL between microglia and neurons [120]. At present, there are still few studies on CTSE and AD. With advancements in the field, the interplay between CTSE and AD, as well as the possible mechanism(s), is now better understood. Nevertheless, rigorous validation is crucial to confirm these insights.

Our current understanding of the role of cathepsin E in AD remains in its early stages. However, it offers a promising avenue for advancing our knowledge of disease mechanisms and potentially leading to the development of novel treatment strategies. Further in-depth validation of these preliminary findings will be essential to substantiate the role of CTSE in AD pathology and its potential as a therapeutic target.

### 4.6 Cathepsin A

Cathepsin A (CTSA) is a serine protease, encoded by the human *CTSA* gene which is located at chromosome region 20q13.12. The gene encodes 480 amino acids. Before it enters the lysosome, the CTSA exists as a zymogen precursor. This inactive form helps prevent uncontrolled proteolysis within the cell. The molecular weight of the precursor structure of CTSA is approximately 54 kDa, which is formed by a disulfide bond between a 32 kDa peptide and a 20 kDa peptide [121, 122]. CTSA is widely found in mammalian tissues, including the liver, kidney, lung, and heart [123]. In addition to its catalytic and protective functions, it is also able to protect β-galactosidase and neuraminidase-1 from proteolysis by lysosomal proteolysis, thus regulating and stabilizing lysosomal activity and function [124, 125]. CTSA-associated arteriopathy with stroke and leukoencephalopathy, which is a rare genetic adult small vessel disease of the human brain, is caused by mutations in the *CTSA* gene and is a late feature of slow cognitive dysfunction [126].

Although the precise role of cathepsin A in AD remains unclear, its involvement in lysosomal function and its protective effects on other enzymes suggest potential implications for disease pathology. Given the established role of lysosomal activity in clearing amyloid-beta peptides, a hallmark of AD, CTSA could theoretically influence disease progression by impacting lysosomal function. Further research is necessary to elucidate the relationship between CTSA activity, lysosomal function, and cognitive decline in AD.

### 4.7 Cathepsin L

Cathepsin L (CTSL) is a cysteine protease. Since CTSL was first proposed in 1973, researchers have isolated and purified two different kinds of CTSA from pig kidneys. During the purification process, CTSA was divided into fragments of different molecular weight sizes, which were named CTSL and CTSS. The human *CTSL* gene is located in the chromosome 9 q21-22 region and encodes a 333 amino acid sequence. CTSL also exists as an inactive zymogen precursor that is composed of a signal peptide, a precursor peptide, and a mature peptide. Mature peptides are further degraded by lysosomes to form heavy chains and light chains. The two chains are connected by disulfide bonds. The spatial structure of CTSL consists of an α-helical L-structure domain and a β-folded R-structure domain [127, 128]. Similar to CTSB, the active site of CTSL consists of cysteine 25, histidine 159, and asparagine 175 [127]. CTSL is involved in many diseases, including tumors, atherosclerosis, kidney disease, dissemination and spread of viral infections [21].

It has been reported that CTSL is also involved in the pathogenesis of neurodegenerative diseases, including AD. According to research by Zheng *et al.*, CTSL functions as a β-secretase in the process of Aβ generation, while the inhibition of CTSL can reduce the oligomerization and the formation of Aβ, which delays the course of AD [129]. Cermak S *et al.* discovered that CTSL and CTSB can control lysosomal function, regulate intracellular cholesterol transport, and mediate characteristic AD proteins. Inhibition of CTSL or CTSB leads to lysosomal function damage and decreases intracellular free cholesterol accumulation and Aβ degradation [130]. The integrity of the nuclear laminae, or laminosis, is widely thought to be a new pathophysiological indicator of AD. A recent study suggested that CTSL affects the progression of AD by affecting the nuclear laminae of neurons. When CTSL expression is upregulated, it can induce damage to the nuclear laminae through cleavage of the nuclear lamin B1 and invagination of the nuclear laminae, leading to chromatin recombination and protein modification. Notably, administration of a CTSL inhibitor could alleviate this phenomenon[131]. Collectively, these findings suggest that CTSL represents a potential target for the treatment of AD.

### 4.8 Cathepsin G

Cathepsin G (CTSG) is also a member of the cathepsin family and is a serine protease. The human *CTSG* gene is located in the q11.2 region of chromosome 14 and has a full-length of 2.7 kb. CTSG consists of five exons and four introns and encodes 255 amino acids sequence. It is found mainly on neutrophils and neutrophils. Recent studies have shown that CTSG is also present in neutrophil extracellular traps and human urine exocrine bodies [132, 133]. CTSG is involved in various functions, including the regulation of inflammation. It can remove pathogens by regulating chemokines, cytokines, and cell surface receptors [132]. In addition, CTSG is a constituent protein of neutrophil granules. Increasing evidence suggests that the inflammatory response caused by neutrophils is involved in AD [134]. It has been suggested that neutrophils can cross the blood-brain barrier to enter the brains of AD model mice. Besides, in the brains of AD patients, Aβ accumulation is accompanied by the infiltration of neutrophils, resulting in neuronal damage and cognitive decline, which can be rescued by CTSG [135]. Moreover, the binding of CTSG to Aβ and advanced glycation end products is involved in the pathogenesis of AD. The main functions of CTSG include cleaving Aβ 1-42, inhibiting oligomers, and preventing Aβ from binding to advanced glycation end products [136]. Whether neutrophil granule proteins, particularly CTSG, can mitigate the progression of AD in cases with neutrophil involvement remains to be elucidated through further investigations.

### 4.9 Cathepsin X

Cathepsin X (CTSX) is a cysteine protease encoded by the cathepsin Z (*CTSZ*) gene. The human *CTSX* gene was first isolated from a human ovarian cDNA library, which is located in the q13 region of chromosome 20 and contains an open reading frame of 912 nucleotides encoding the 303 amino acid precursor protein [137]. The molecular weight of natural CTSX is 53 kDa. Like other cathepsin, CTSX is also synthesized with an inactive proenzyme, which is converted into mature CTSX by CTSE. CTSX is highly expressed in immune cells, including monocytes, T lymphocytes, macrophages, and dendritic cells. It is also expressed in nerve cells, such as neurons in healthy persons and AD patients in neurons and glial cells in patients with multiple sclerosis. This diverse tissue distribution suggests CTSX involvement in processes beyond immune regulation, potentially influencing neuronal function and contributing to disease mechanisms [138-140]. According to the work of PISAR A *et al.*, LPS treatment activates microglia, which simultaneously upregulates the expression of CTSX. Treatment with the CTSX inhibitor AMS36 was found to inhibit microglial activation, microglial death, and apoptosis [141]. In addition, CTSX was found to be upregulated in microglia surrounding amyloid plaques in AD model mice [140]. Thygsenc *et al.* analyzed the proteomics of hippocampal and central nervous system myeloid cell samples from the 9-12-month-old APP/PS1AD model mice injected with LPS daily and found that four proteins, including CTSX, APP, APOE, and CLU, were upregulated significantly compared to the control group. Besides, CTSX exhibited changes in immune response patterns in postmortem cortical tissues of AD patients [142].

While the precise mechanisms underlying CTSX's influence on AD pathology remain elusive, current evidence suggests its involvement in neuroinflammation, microglial activation, and the central nervous system's immune response. As research advances, CTSX may hold promise as a novel therapeutic target for AD treatment.

## 5. DISCUSSION AND PERSPECTIVE

AD represents a multifaceted neurodegenerative disorder characterized by the progressive deterioration of cognitive functions [143]. The pathophysiological hallmarks include the aggregation of misfolded proteins, such as Aβ plaques and tau tangles, leading to synaptic dysfunction and neuronal loss [144-146]. Despite extensive research, the precise etiology of AD remains elusive. It is widely thought that genetic, environmental, and age-related factors contribute to its onset and progression [147, 148].

The cathepsin family plays a significant role in AD, impacting key pathological processes such as Aβ deposition, hyperphosphorylation of Tau protein, and regulation of inflammatory responses (Fig. 5). Several cathepsins, including CTSB, CTSD, CTSE, CTSS, CTSL, and CTSG, are implicated in Aβ metabolism, influencing its accumulation in the brain. CTSB can modulate Aβ degradation [79], while CTSD is associated with the formation of Aβ plaques [91]. CTSE is involved in regulating β-secretase expression, which in turn affects Aβ formation and deposition [120]. Notably, CTSS reportedly decomposes APP at a rate 1170 times faster than the β-secreting enzyme [114]. Additionally, CTSL and CTSG may both function as β-secretases in Aβ generation, while inhibiting CTSL can reduce Aβ oligomerization and formation [130, 135]. Beyond Aβ, cathepsins, including CTSD and CTSE, may influence Tau protein phosphorylation through direct or indirect mechanisms, contributing to the formation of NFTs. For instance, documented cleavage sites of CTSD on Tau proteins suggest its significant role in degrading nerve fiber junctions [92]. Similarly, CTSE significantly increases Tau protein phosphorylation at multiple residues [120]. Moreover, cathepsins, including CTSG [132], CTSS [111], CTSH [104], and CTSX [138], are linked to the activation of inflammation and immune responses in the AD brain. These cathepsins modulate the function of immune cells and the release of inflammatory mediators. This can involve activating microglia and promoting the secretion of inflammatory cytokines, ultimately exacerbating brain inflammation. In summary, cathepsins exert multifaceted effects on the pathophysiological processes of Alzheimer's disease. These effects include, but are not limited to, Aβ metabolism, Tau protein phosphorylation, and regulation of inflammatory responses.

**Figure 6.**
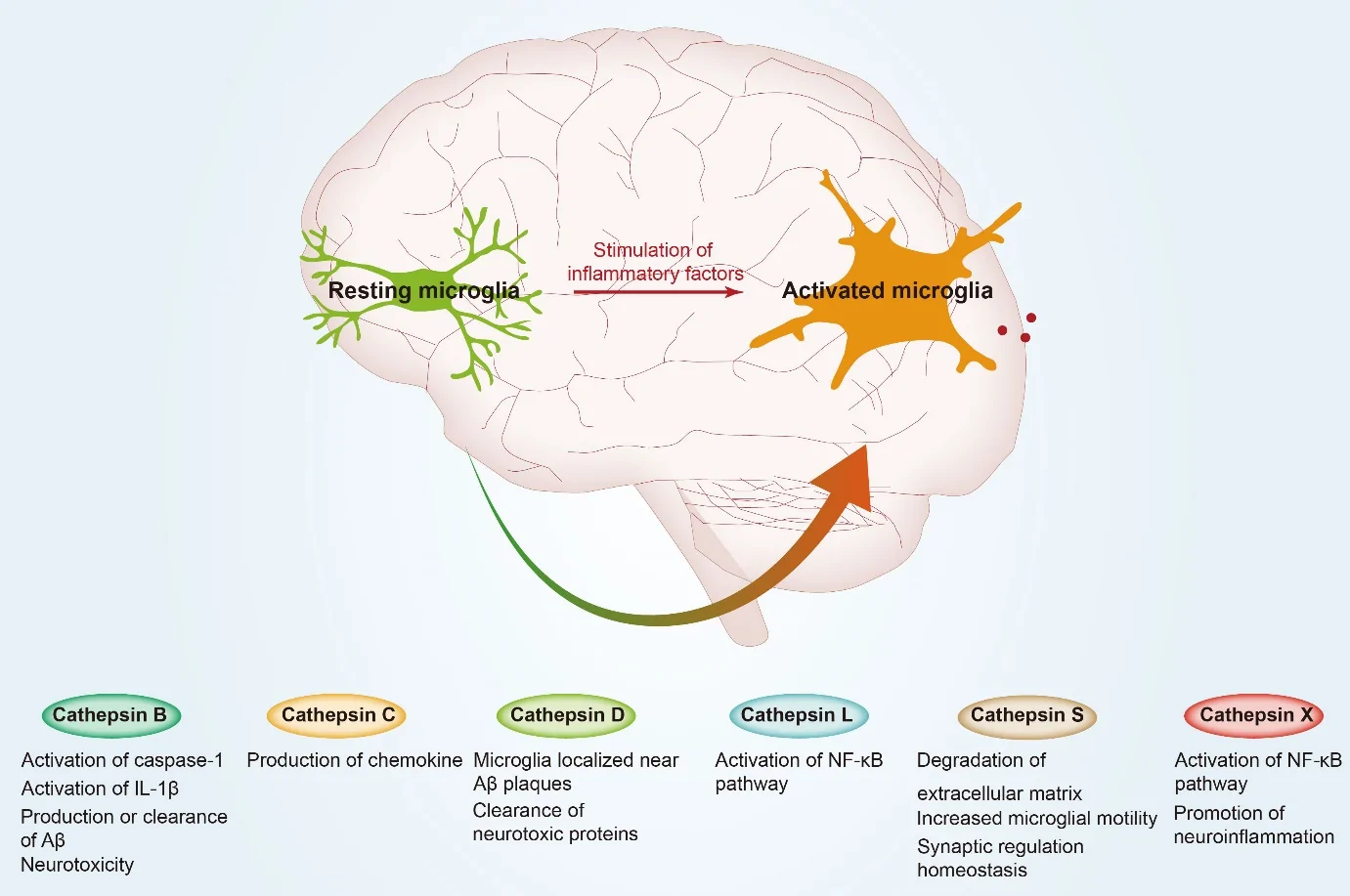
**The involvement of cathepsins in neuroinflammation**. Microglia, the resident immune cells of the central nervous system, exhibit dynamic phenotypic transitions. Exposure to diverse inflammatory factors induces their switch from a quiescent (resting) state to an activated phenotype characterized by cathepsin release. Among these proteases, extracellular cathepsin B stands out for its pro-inflammatory effects through caspase-1 and IL-1β activation. Moreover, it modulates the production and clearance of amyloid-β (Aβ), a hallmark of neurodegenerative diseases, influencing its neurotoxicity. Cathepsin C contributes to inflammation by promoting chemokine production. Notably, cathepsin D, secreted by microglia adjacent to Aβ plaques, plays a crucial role in the clearance of these neurotoxic proteins. Finally, cathepsins L and X further amplify inflammation via NF-κB pathway activation. Ultimately, cathepsin S can degrade the extracellular matrix and increase the motility of microglia, which are important for synaptic homeostasis.

Our review of the cathepsin family's potential as a therapeutic target for AD highlights promising avenues for further research and development. Notably, dysregulation of cathepsin activity has been implicated in AD pathogenesis, influencing the activation of microglia and astrocytes and contributing to the accumulation of Aβ and tau [149]. Besides, the classification, biological features, and physiological functions of the cathepsin family have been thoroughly elucidated in this review. Elucidating the intricate roles of cathepsins in cellular processes, such as protein degradation and immune regulation, provides crucial insights into their potential contribution to the pathogenesis of AD. Notably, the influence of cathepsins on the activation of microglia and astrocytes, the resident immune cells in the brain, suggests a pivotal role in modulating the neuroinflammatory response associated with AD [15, 120, 150, 151]. The dysregulation of cathepsin activity may lead to the accumulation of misfolded proteins, particularly Aβ and tau, hallmark features of AD pathology [152]. This finding reinforces the hypothesis that cathepsins play a crucial role in the proteostasis network of the brain, and their dysfunction may contribute to the vicious cycle of protein aggregation and neuronal damage in AD [153]. Moreover, the potential for dysregulated cathepsin activity to promote chronic inflammation in the brain raises important questions about the broader implications of targeting cathepsins as a therapeutic strategy. While dampening neuroinflammation is a desirable goal for AD treatment, it is crucial to consider the delicate balance of immune responses in the brain and avoid inadvertently disrupting essential functions of the immune system (Fig. 6).

Cathepsins, with their diverse contributions to AD pathology, hold promise as therapeutic targets, but significant knowledge gaps remain. Firstly, a deeper understanding of individual cathepsin roles in Aβ production, tau hyperphosphorylation, and neuroinflammation is crucial. Elucidating these precise mechanisms will require further investigation. Secondly, the potential of cathepsin inhibitors as therapeutic agents in AD needs exploration. Research should focus on developing selective inhibitors and evaluating their impact on disease progression in preclinical models and clinical trials. Thirdly, the correlation between cathepsin levels, disease severity, and cognitive decline in patients remains poorly understood. Prospective studies monitoring cathepsin levels alongside disease progression could provide valuable insights. Additionally, investigating genetic variations in cathepsin genes and their association with AD risk could uncover novel genetic factors. Furthermore, research is needed to understand how cathepsins interact with other cellular pathways implicated in AD, such as endosomal-lysosomal function and autophagy. The emerging role of neuroinflammation in AD necessitates further investigation of how cathepsins modulate the central nervous system's inflammatory response and contribute to neuronal damage. Finally, translating findings from cellular and animal models to human disease is critical. This necessitates evaluating cathepsin-targeted therapies in clinical trials to assess their efficacy and safety in human subjects. By addressing these research gaps, we can unlock the full potential of cathepsins as therapeutic targets for AD.

Treatment strategies could include the development of cathepsin inhibitors to reduce Aβ production or facilitate its degradation, mitigate Tau protein hyperphosphorylation, and modulate inflammatory responses. Additionally, targeting cathepsins at the molecular level using gene editing technologies like CRISPR/Cas9 or RNA interference (RNAi) techniques opens up new avenues for treatment. There is also the possibility of developing multitarget drugs that simultaneously target cathepsins and other pivotal targets in AD.

In addition, the potential of cathepsins as biomarkers for early diagnosis and disease progression in AD warrants further evaluation, involving systematic studies of cathepsin expression and activity at different stages of the disease, as well as their correlations with AD symptoms and progression. The development of novel animal models represents a critical step towards achieving a more precise *in vivo* recapitulation of the AD pathological milieu. These models would facilitate a more granular investigation into the functional role of cathepsins within the context of AD pathogenesis. Furthermore, such models would serve as invaluable tools for evaluating the impact of genetic manipulations targeting cathepsins on the disease process, ultimately paving the way for the development of novel therapeutic strategies.

The identification of cathepsins as potential therapeutic targets opens new avenues for the development of targeted interventions for AD. Strategies aimed at modulating cathepsin activity could offer a novel approach to mitigate the pathological processes underlying AD. Future studies should focus on the specific mechanisms by which cathepsins contribute to neuroinflammation and protein aggregation, paving the way for the design of precise and effective therapeutic interventions. However, research targeting cathepsins as therapeutic agents for AD is challenging, warranting a more comprehensive understanding of their role in AD. Besides, the development of cathepsin inhibitors necessitates a rigorous evaluation of potential adverse effects and toxicity, particularly due to their critical roles in normal physiological processes. Finally, identifying which members of the cathepsin family are most promising as therapeutic targets and how to precisely modulate their activity are key areas for future research.

**Figure 7.**
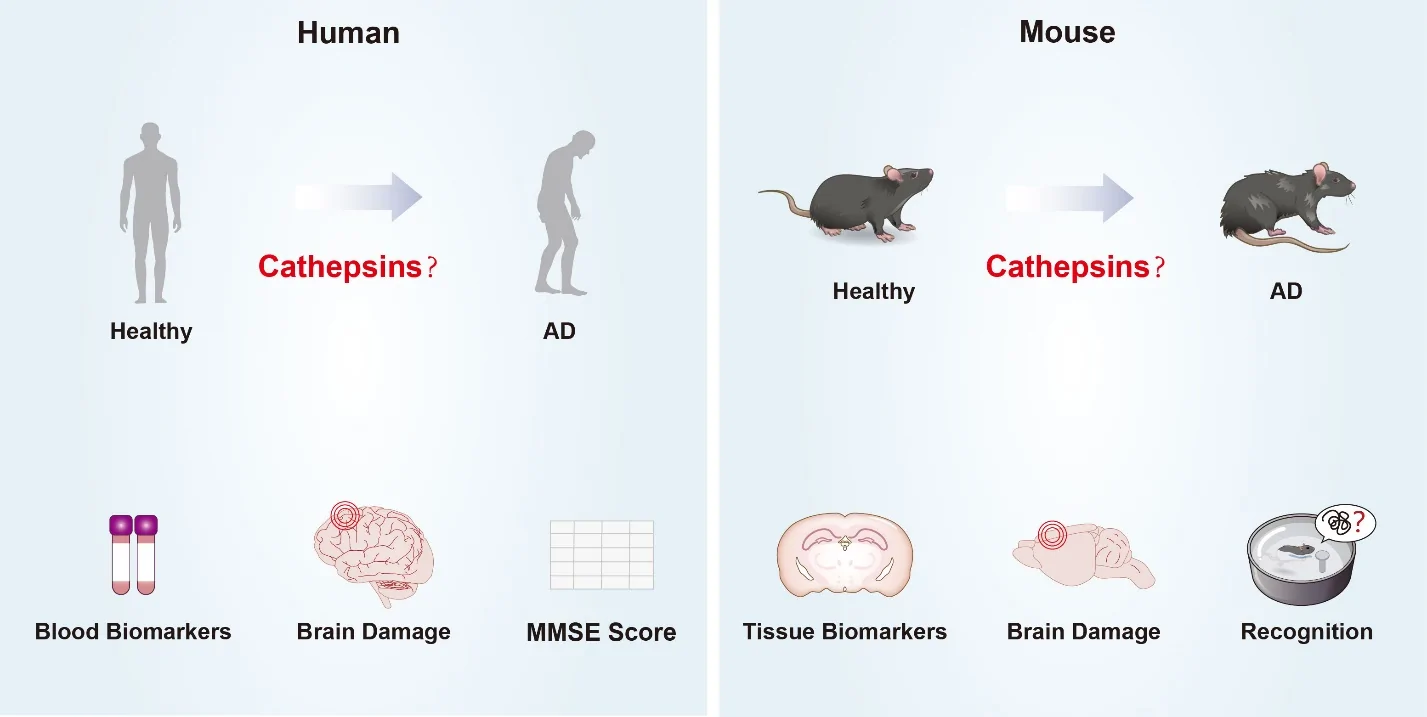
**Graphic summary**. Our review illustrated that cathepsins exerted vital roles in AD progression both in humans and mice, while dysfunction of cathepsins would lead to brain damage and cognition deficits, which suggested that cathepsins could be a potential targets and biomarkers for early diagnosis and later drug development and treatment for AD.

## 6. CONCLUSIONS

In conclusion, our review has expounded on the multifaceted roles of several cathepsins, previously investigated in the context of AD pathogenesis both in humans and mice. We have demonstrated their diverse regulatory abilities in AD progression, encompassing functions such as modulating the neuroimmune response, facilitating Aβ degradation, and influencing tau hyperphosphorylation, all of which are critically contribute to the brain damage and cognitive dysfunction. While our review has encompassed a wide spectrum of cathepsins, it is evident that some members of this essential class of lysosomal enzymes warrant further investigation. As research advances, the intricate interplay between cathepsins and AD is becoming increasingly unraveled, suggesting their potential as promising targets and biomarkers for early diagnosis and later drug development and treatments for AD (Fig. 7).
